# Designing a Self-Guided Digital Intervention for Self-Management of Shoulder Pain in People Living With Spinal Cord Injury: Tutorial on Using a Person-Based Approach

**DOI:** 10.2196/66678

**Published:** 2026-01-05

**Authors:** Verna Stavric, Nicola L Saywell, Nicola M Kayes

**Affiliations:** 1 Department of Physiotherapy, School of Allied Health, Auckland University of Technology, Private Bag 92006, Auckland, 1142, New Zealand, 64 9-921-9666 ext 7060; 2 Centre for Person Centred Research, Auckland University of Technology, Auckland, New Zealand

**Keywords:** person-based approach, self-guided, intervention design, behavioral analysis, spinal cord injury, shoulder pain, self-management, mHealth, mobile apps, smartphones, digital health, digital interventions

## Abstract

Shoulder pain is prevalent in people living with spinal cord injury. Technology and digital rehabilitation tools are increasingly available, but this has not yet included the provision of a self-guided exercise intervention focused on managing shoulder pain for people living with spinal cord injury. We drew on the person-based approach (PBA) to intervention development to design a Shoulder Pain Intervention delivered over the interNet (SPIN) to address this gap. However, in preparation for the design process, we found very few published examples of how the PBA had been operationalized. The aim of this paper is to provide a detailed explanation of our approach and how we operationalized the PBA in the design of SPIN to maximize relevance and engagement. Our design process followed the key PBA steps, combining additional evidence and theoretical components. Each step ensured that guiding principles were formulated and followed to maximize the probability that SPIN would be fit for purpose. We followed 3 steps: (1) we drew on themes from preparatory research (existing and primary) to identify the key behavioral issues, needs and challenges, and existing features to form the basis of SPIN design; (2) we formatted guiding principles that included articulating specific design objectives to provide a framework to identify system requirements; and (3) we selected and refined intervention features using existing literature, behavioral theory, and tools such as the “Behaviour Change Wheel.” We have designed SPIN by incorporating a deep understanding of the users’ needs and best available evidence to maximize engagement and positive outcomes. In this paper, we have made clear how we operationalized the PBA phases, including how existing evidence, theory, tools, and methods were leveraged to support the PBA process. In explicating our process, we have provided a blueprint to guide future researchers using this approach.

## Background

### Overview

Shoulder pain is common in wheelchair users living with spinal cord injury (SCI) [1,2]. A lesion to the spinal cord can result in loss of innervation to muscles of the trunk and lower limbs. Consequently, many people living with SCI (pwSCI) rely on their upper extremities not only for performance of daily activities but also for locomotion. Shoulder pain can have a significant impact on their activity, reducing mobility, independence, and quality of life [1-5]. Digital and web-based interventions have increasingly been offered to pwSCI to promote exercise and physical activity [6-9]. These interventions minimize barriers to rehabilitation to address many health concerns, including managing their shoulder pain. Previous authors have found that in the general population, digital or web-based interventions can produce positive effects in various outcomes, such as physical activity [10,11].

Technology-supported exercise interventions for pwSCI with persistent shoulder pain are currently available, but they have some limitations. They either require ongoing input and monitoring from a clinician [12-15] or provide general self-management advice [13] but without enough guidance to allow for clear and structured exercise progression specifically for shoulder pain. Self-guided digital exercise interventions have been successfully implemented for people with knee osteoarthritis [16,17], dizziness [18,19], and breast cancer [20] and may be a viable option for pwSCI. Our recent systematic review and meta-analysis of self-guided digital physical activity and exercise interventions demonstrated positive effects on physical activity at both short- and longer-term follow-up, in people living with chronic conditions [21]. We also found that interventions that used behavioral strategies and were underpinned by a theoretical framework were more effective. This suggests that self-guided digital interventions have the potential to support pwSCI to manage their shoulder pain, but that the intervention would need to be designed systematically and intentionally.

We have designed Shoulder Pain Intervention delivered over the interNet (SPIN) as a self-guided digital intervention to give pwSCI who experience shoulder pain the ability to access and progress evidence-based exercises. The intervention guides pwSCI to monitor symptoms and improvement [22] to promote autonomy in the management of their condition. The aim of SPIN is to be an engaging program that is responsive to the needs of pwSCI who have shoulder pain.

To achieve this, we were guided by the person-based approach (PBA) in the design of SPIN [23]. The PBA follows 4 iterative phases of intervention development that include (1) planning which seeks a deep understanding of the perspectives and psychosocial context of potential users through iterative qualitative research, (2) design based on guiding principles that have been created from insights from the first phase, (3) development and refinements which are made through iterative user feedback, and (4) trialing to evaluate the effectiveness on outcomes and impact on behavior change to make any necessary adjustments. Due to its focus on the development of digital behavior change interventions, the intent and purpose of PBA align well with adjacent behavior change theory and tools such as the COM-B [24], “Behaviour Change Wheel” [25], and behavioral analysis [26]. Furthermore, the PBA process is sufficiently flexible to enable the use of these (and other) tools to achieve the aims and purpose of a given phase. Integrating behavioral science theory and evidence while keeping users’ needs and contexts in focus has been found to maximize engagement and effectiveness of interventions [18,25,27-31]. This tutorial focuses on the first 2 PBA phases of planning and design. See Table 1 for an example of how our study was mapped onto the PBA.

**Table 1.** Mapping of person-based approach phases onto Shoulder Pain Intervention delivered over the Internet design.

PBA^a^ description	Phase	This study	
		Purpose	Planned outcome
Use of primary and secondary *qualitative evidence* to understand users’ behavioral and psychosocial needs and challenges in using the intervention	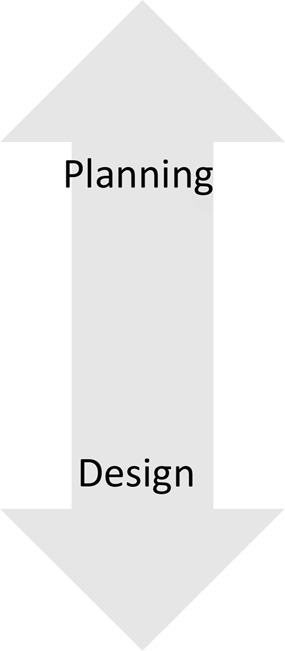	To determine *factors that need to be included* to *encourage or facilitate engagement* with this self-guided web-based exercise intervention	A rich description of key *needs, challenges, and facilitators* of engagement in web-based tools and exercise for people living with SCI^b^ who experience shoulder pain to *underpin the design phase’s guiding principles and features*
Formulation of *key guiding principles* that capture the main intervention objectives as identified in the planning phase and that are continuously referred to throughout the development of the intervention		To design an *evidence-based, self-guided, web-based* intervention Exercise, behavioral support, and self-guided components to be included within the intervention features	Intervention design objectives Intervention features First iteration of SPIN^c^ prototype

a PBA: person-based approach.

b SCI: spinal cord injury.

c SPIN: Shoulder Pain Intervention delivered over the interNet.

We drew heavily on the existing PBA literature during the planning and designing stages of SPIN. However, the lack of access to detailed examples of how the PBA has been operationalized in practice made it challenging to translate the principles of this approach into reality. This is not a unique problem. Duncan and colleagues [32] noted that published work on the development of an intervention is frequently sparse because it is often included in the same publication as the reporting of a pilot or feasibility study.

### Aim

The aim of this paper is to make clear how the principles of the PBA were operationalized in intervention design and the development of SPIN. We have illustrated our use of the PBA framework by outlining the detailed and explicit steps involved in the translation of the evidence, theory, and person-based recommendations into intervention design. In doing so, we have built on the existing methodological framework and enabled others to draw on this approach in future intervention design and development.

## Methods and Outcomes

### Overview

**Figure 1.**
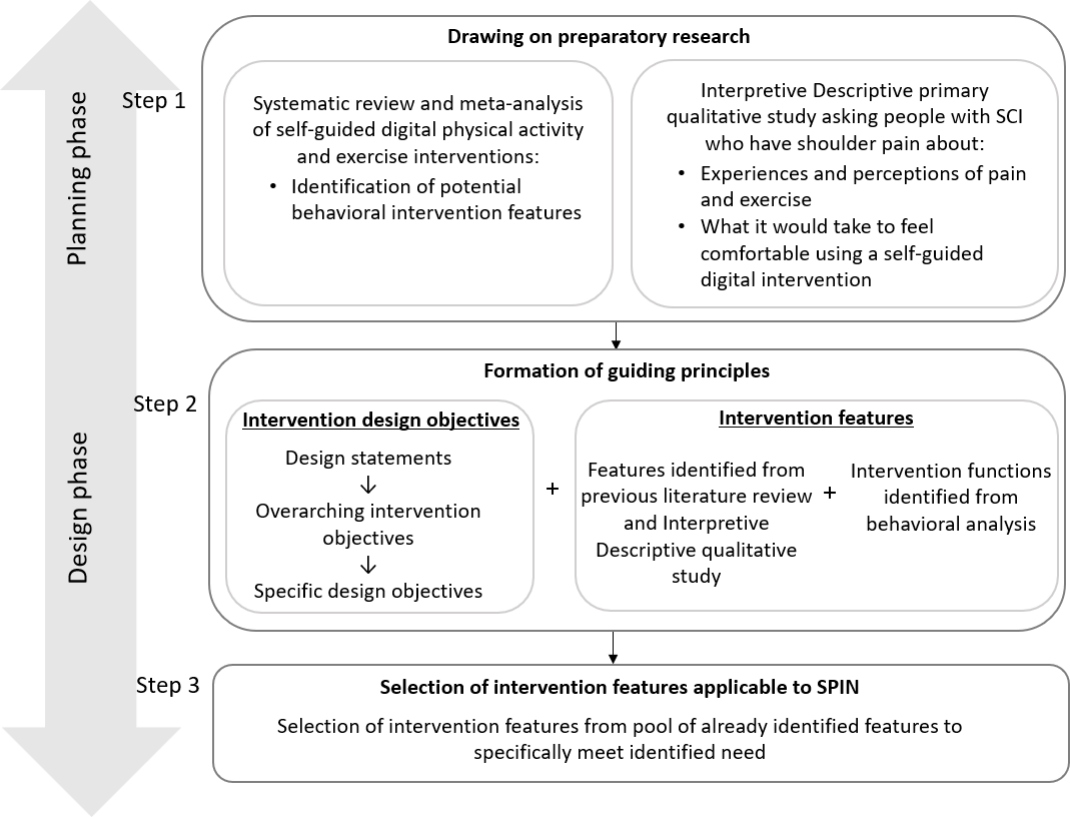
Overview of Shoulder Pain Intervention delivered over the Internet design steps and components. SCI: spinal cord injury; SPIN: Shoulder Pain Intervention delivered over the interNet.

The planning and design phases of the PBA are described below, along with an overview of how they were operationalized in the design of SPIN. Figure 1 provides an overview of the SPIN design process and the components involved. Each step and its subsequent outcome have been described in detail in the sections that follow.

### Step 1: Drawing on Preparatory Research

#### Methods

This initial phase of the PBA draws on qualitative research, including interviews and focus groups, to gather in-depth insights into the psychological, social, and emotional factors that influence the users’ behavior. The goal is to identify the underlying motivations, beliefs, and barriers that may affect engagement with health interventions [23]. In the context of SPIN, this preparatory research included: (1) a systematic review and meta-analysis investigating the effectiveness of self-guided digital physical activity and exercise interventions [21] and (2) an Interpretive Descriptive qualitative study exploring the perceptions of pwSCI who have shoulder pain, on the use of a self-guided digital intervention to help them manage their shoulder pain [33].

#### Outcome

The review identified several self-guided digital physical activity and exercise interventions. Data extraction included identifying discrete intervention features and categorizing them using a purpose-built template (Multimedia Appendix 1), based on a synthesis of key literature [27,34-40]. Using this template, we extracted possible behavioral intervention features relating to qualities such as customizability, the provision of instruction, feedback and monitoring, tailoring, reminders and prompts, goals and planning, social support, and rewards and threats. We also noted the success of interventions using features that supported behavior, particularly self-regulation. This informed an initial pool of possible intervention features for SPIN that were reviewed later in Step 2.

The Interpretive Descriptive qualitative study identified themes that represented an evaluative process pwSCI go through when considering using a self-guided digital exercise intervention: *Should I use it?,* whether I believe it will work for me right now; *Can I use it?,* whether I can operate the intervention competently and confidently; and *Will I use it?,* whether it will be responsive to my unique needs and keep me coming back. These formed the basis of the design statements in Step 2.

Conceptual representations of possible behavioral intervention features identified from the review were used as probes and images during data collection in the Interpretive Descriptive qualitative study. These were used to prompt discussion about what could help pwSCI to engage in a self-guided digital intervention. The pwSCI discussed ways in which these concepts and specific features may support them. These perspectives were extracted from the audio recordings and tabulated to support the identification of behavioral intervention features in Step 2.

### Step 2: Formation of Guiding Principles

#### Overview

The guiding principles in the PBA are formulated by synthesizing key insights from the planning phase (Step 1) into intervention design objectives and corresponding intervention features that address users’ specific needs, preferences, and behavioral barriers [23]. Yardley and colleagues [23] contend that staying true to the identified needs of the people who will use the intervention, throughout the design process, increases intervention relevance, engagement, and effectiveness. In our design of SPIN, we followed several stages to ensure the key context-specific behavioral needs and challenges identified in the Interpretive Descriptive qualitative study remained the focal point during intervention design.

#### Intervention Design Objectives

Yardley et al [23] suggest generating intervention design objectives to support the creation of the guiding principles but do not expand on how these may be identified. Below, we describe the method we followed to produce intervention design objectives through the creation of design statements, overarching intervention objectives, and specific intervention design objectives.

#### Design Statements

##### Methods

We created design statements by using the 3 themes constructed in the Interpretive Descriptive qualitative study. We first reframed each theme into a design statement, giving consideration to how each could be reflected in the design of the intervention. To do this, we reworded the themes to move from a question (*Should I use it?*) into a design statement (*I should use it if…*) and then added conditions applicable to each design statement. Each condition reflected key elements from the qualitative findings, resulting in person-centered conditions to be met in the design process. This process provided depth and context to inform the design of SPIN and ensured the next step would be underpinned by the perspectives of the future users of the intervention, in this case, pwSCI.

##### Outcome

The Interpretive Descriptive qualitative study themes, design statements, and key conditions for success are presented in Table 2.

**Table 2.** Translation of themes to design statements and conditions of success.

Interpretive Descriptive qualitative study theme	Reframed to: design statements	Conditions for success
*Should I use it?*	I should use it if:	I believe it will work for me There is evidence of credibility There is a clear indication that it is suitable for me It resonates with my current attitude toward exercise, support situation
*Can I use it?*	I can use it if:	I can use it competently I can use it confidently It can be tailored and adapted to my unique needs I can use it safely, without causing more harm I have the belief that I could use it, given the resources and capacity I have I have the right support to use it
*Will I use it?*	I will use it if:	It is responsive to my unique needs It encourages me to progress when I am ready I feel supported to use it I can see progress as a consequence of using it It keeps me coming back

### Overarching Intervention Objectives

#### Methods

Next, we articulated the overarching intervention objectives. Succinctly describing the intervention objectives allows a snapshot of the key characteristics of the intervention [23]. We, therefore, clearly articulated how SPIN is distinctive and different from other interventions, reflecting the specific behavioral issues, needs, and challenges it must address.

We developed the intervention objectives iteratively, repeatedly revising the wording with reference to the original research question and design statements, and with input from the research team and stakeholders. Stakeholders included pwSCI, a clinician with experience in SCI rehabilitation, a clinician who was also a pwSCI and a representative of a relevant nongovernmental organization. Each iteration strived to reflect the essence of the needs expressed by the participants with wording that represented what ideal uptake and use of this self-guided digital exercise intervention could look like. The overarching intervention objectives were then used as a reference point for later design and development phases.

#### Outcome

Referring to the design statements and overall research aim, the overarching intervention objectives for SPIN were to:

1. Be tailored to users’ specific and unique needs so they can relate to it and trust it and so that it can be responsive to their changing needs while using SPIN; and
2. Enable users to use it competently and confidently within their capabilities and support systems in a way that is safe and motivating.

### Specific Design Objectives

#### Methods

Once the overarching intervention objectives were formulated, we created the specific design objectives underpinned by the design statements. We developed a working definition, incorporating the key conditions for success for each specific design objective, to ensure clarity in interpretation. These were then reviewed against the overarching intervention objectives, making sure they supported the overall objectives of SPIN. We continued to refine them as the design process progressed, during our planned discussion forums.

#### Outcome

Tables3^5^ each refer to a different theme. Specific design objectives and working definitions are presented in the first 2 columns; intervention functions and features are discussed in later sections.

**Table 3.** Guiding principles from the theme *Should I use it?*.

Design objectives that address identified needs, issues, and challenges	Working definition	Intervention functions	Intervention features that address the design objectives
To help users relate to and trust the program	The program will give users confidence in the source, message, and value of the program. The program is credible and legitimate and promotes trust.	Education Training Modeling Enablement Persuasion	Development team details (names, credentials, and contact info) Endorsements *Testimonials* (source matching for social comparison) Evidence for shoulder pain exercises How user data will be used or stored Professional polished interface and function
To reassure users it will be clear who the program is suitable for, giving users confidence that the program is right for them and at what stage it is right for them	The program will guide users through a process to be able to screen for and identify if they are suitable to use the intervention and to promote trust and confidence that this is a safe and robust process.	Education Training Modeling Enablement Persuasion	Screening questionnaire/questions (that will exclude those unsuitable) Monitoring questions at each exercise event and tracking this information *FAQ^a^ section* Contact information for the team
To provide a sense of potential that it will work for them	The program will help users identify with it and the potential that it may have for them, in their current situation.	Education Training Modeling Enablement Persuasion	*Testimonials* (image with text, video, and quotes) of people in different “stages” of readiness or different situations. FAQ section addressing suitability of different situations “Is this right for me?” or “How do I know this is right for me?” or “Questions I can ask to make sure this is right for me?”

a FAQ: frequently asked question.

**Table 4.** Guiding principles from theme *Can I use it?*.

Design objectives that address identified needs, issues, and challenges	Working definition	Intervention functions	Intervention features that address the design objectives
To promote a sense of safety when using the program	The program will ensure exercises are at the appropriate difficulty level and will be responsive to changes in user presentation to ensure that they don’t significantly aggravate shoulder symptoms.	Training Environmental restructuring Modeling Enablement	*Monitoring and tracking of shoulder pain* and exercise difficulty Exercise selection based on user responses and a priori rules Program-generated advice based on user responses, such as acknowledging concerns, referral to *FAQ^a^*, evidence, health care provider
To promote user competence	The program will be easy to use by a range of users and in a range of circumstances, giving them a sense of confidence when using it in the context of their unique life situation.	Training Environmental restructuring Modeling Enablement	Language at an appropriate reading level Layout is clear and simple Font size and buttons are large for reduced hand function Minimal scrolling and clicking Consistent screen layout Clear signposts Logical interface Exercises presented in video and audio formats by pwSCI Exercises presented in step-by-step processes Exercises are planned to fit in with daily routine and normal digital device use Tunneling of information (releasing information in small amounts, as the user progresses through “right amount, at the right time”) Graded goal setting, implementation planning *Tailored* and action feedback based on tracking Praise for success Advice or support if not yet succeeded Digital use guidance when needed (help link)
To promote user autonomy	The program will give users a sense of control and ownership over the program and their progress through the program.	Training Environmental restructuring Modeling Enablement	*Offering choice where possible: tailoring functions* in exposure matching-timing, intensity (when and how often) *Reminders* Excercise selection, timing of exercise Intervention delivery Tunneling of options into the most common choices Suggestions or options for different situations

a FAQ: frequently asked question.

**Table 5.** Guiding principles from the theme *Will I use it?*.

Design objectives that address identified needs, issues, and challenges	Working definition	Intervention functions	Intervention features that address the design objectives
To promote a positive emotional experience	The program will incorporate positive autonomy-supportive language that invites, informs, and supports users to work through the program.	Training Environmental restructuring Enablement Modeling Education Persuasion Incentivization	Use of *positive language and tone* in inviting users to decide for themselves “some find it helpful.” Use of anecdotes to describe examples of success, decision-making Acknowledging and addressing concerns about using the program, such as pain or carer support Using *FAQ^a^ section* Use of useful/interesting/relevant/personal reminders Positive or encouraging wording on feedback on progress toward the goal
To promote a sense of relatedness	The program will be relevant to the user by using communication and wording that is tailored to their self-identified preferences and personalized to their unique circumstances.	Training Environmental restructuring Enablement Modeling Education Persuasion Incentivization	Feedback as above (and that is immediately reciprocated when interacting with the intervention) Competition with others, and/or Cooperation with others Social connection through the program’s grouping Initial “getting to know you” questionnaire to help with *tailoring ingredients* Personalization: (1) identification (including username in correspondence), (2) raising expectation (including relevant information in correspondence that is based on users’ responses to questions/input), and (3) contextualization (*wording, examples that are relevant to user*-exercises relevant for tetra vs para) *Reminders* *Testimonials* Self-identified support
To help users maintain their exercise over the 12 weeks	The program will use a variety of strategies and features to encourage and support users to maintain engagement in their exercise for the duration of the program.	Training Environmental restructuring Enablement Modeling Education Persuasion Incentivization	Rewards (points or similar)/competition Goal setting Action planning Communication that is *positive***,** immediate, and useful and *tailored*
To promote a sense of accountability	The program will provide features that encourage the user to return to the program and to continue with the exercises.	Training Environmental restructuring Enablement Modeling Education Persuasion Incentivization	Competition with others or with self Support from others Communication that is *positive*, immediate, and useful and *tailored* Communication that is personalized Rewards that are only released upon completion of a certain amount of exercise
To promote a sense of progree and engagement	The program will enable the user to understand their progress through a clear and simple tracking feature. This will be done in a way that encourages further progress and ongoing engagement with the exercise intervention	Training Environmental restructuring Enablement Modeling Education Persuasion Incentivization	Feedback and tracking Choice in exercise selection Personalization *Tailoring*

a FAQ: frequently asked question.

### Intervention Features

**Figure 2.**
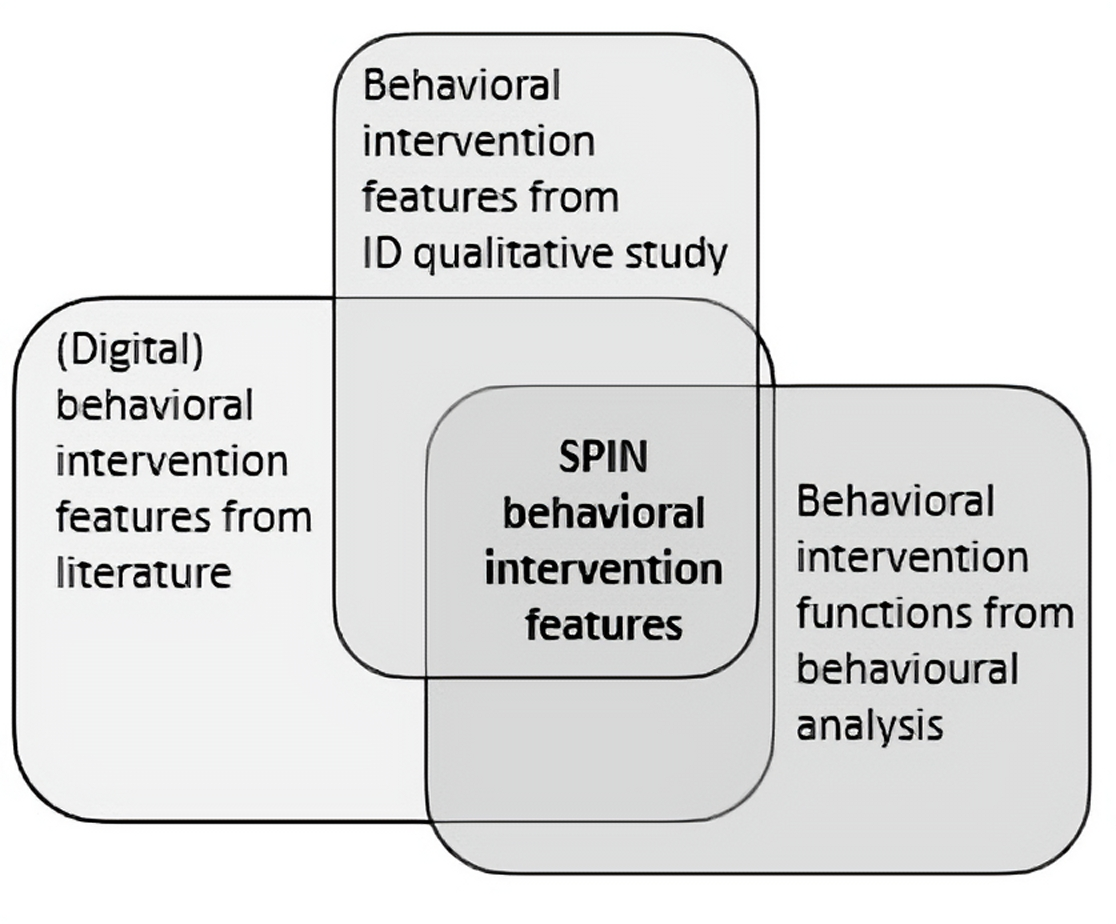
Layers of evidence that informed Shoulder Pain Intervention delivered over the Interjet’s intervention features. ID: Interpretive Descriptive; SPIN: Shoulder Pain Intervention delivered over the interNet.

In the PBA, the guiding principles inform the intervention features by providing a framework for selecting and shaping features that directly support the specific design objectives, and to improve resonance, engagement, and acceptability of an intervention [23]. A range of evidence informed the selection of behavioral intervention features: (1) in our review, we identified a range of features used in digital interventions that have been associated with better health-related outcomes [27,34-36]; (2) we identified possible behavioral intervention design features from our Interpretive Descriptive qualitative study [33]; and (3) we identified behavioral “intervention functions” we were trying to achieve using a behavioral analysis as per Michie and colleagues’ framework [26]. We then mapped these to the most relevant intervention features. Figure 2 represents the layers of evidence that informed SPIN’s intervention features. We will describe each of these in detail below.

#### Identifying Behavioral Intervention Features From Previous Literature Review and the Interpretive Descriptive Qualitative Study

##### Methods

In Step 1, we had earlier identified potential behavioral intervention features for self-guided interventions that were identified from our systematic review and meta-analysis, using the specifically developed template, drawing from the CONSORT-EHEALTH checklist (V.1.6.1) [38]. See Multimedia Appendix 1 for a sample of our template showing sections used to record behavioral intervention features. For this current stage of the SPIN design, we also reviewed intervention features of publications that missed the strict inclusion criteria of the systematic review and meta-analysis but addressed digital delivery of physical activity or exercise intervention for possible relevant behavioral intervention features. We then integrated the data on specific features collected from our Interpretive Descriptive qualitative study. These data were categorized by proposed purpose and function and then mapped against the specific design objectives.

##### Outcome

There was overlap, resulting in some features identified as addressing more than one design objective. Many of the studies in the systematic review included digital behavioral intervention features that involved instruction on exercise or physical activity performance, self-monitoring of the exercise or physical activity behavior, goals and planning, and prompting. The results of the Interpretive Descriptive qualitative study and other reviewed literature suggested additional behavioral intervention features. Table 6 presents a summary of the behavioral intervention feature categories that we considered for SPIN, the design objective(s) they are related to, and the supporting evidence.

**Table 6.** Behavioral intervention feature categories supported by systematic review, Interpretive Descriptive qualitative study, and existing literature.

Design objectives	Behavioral intervention feature	Proportion of studies identified in systematic review and meta-analysis (out of 16 studies)	Identified in Interpretive Descriptive qualitative study	Identified in other literature not included in meta-analysis
*Should I use it?*	Ensuring personal relevance	16	✓	Horsch et al [41]
*Should I use it?*/*Can I use it?*	Use of credibility and trust-enhancing features	5	✓	Bossen et al [42]; Oinas-Kukkonen and Harjumaa [43]
*Should I use it?/Will I use it?*	Provision of information about actual users	2	✓	Morrison et al [34]
*Can I use it?*	Allowance of the user to control or adapt features	7	✓	McClure et al [44]
*Can I use it?*	Ensuring ease of use	6	✓	Carter et al [45]; Hurling et al [46]; Webb et al [27]
*Can I use it?*	Provision of information ‘just in time’ and in ‘just the right amount’	9	✓	Oinas-Kukkonen and Harjumaa [43]; Xu et al [47]
*Can I use it?*/*Will I use it?*	Use of goal setting	8	✓	Webb et al [27]; Willett et al [48]; Dugas et al [49]
*Can I use it?*	Use of demonstration of behavior	10	✓	Webb et al [27]
*Can I use it?*	Use of feedback of behavior	10	✓	Webb et al [27]; Dugas et al [49]
*Can I use it?*	Use of tailored feedback	10	✓	Morrison et al [35]; Dugas et al [49]
All	Use of tailoring based on a number of variables	5	✓	Morrison et al [34]; Couper et al [50]; Xu et al [47]; Oinas-Kukkonen and Harjumaa [43]; Figueiras and Neto [51]; Dugas et al [49]
*Can I use it?*/*Will I use it?*	Use of reminders	8	✓	Webb et al [27]; Lin and Wu [52]; Alahäivälä and Oinas-Kukkonen [53]; Dugas et al [49]
*Can I use it?*/*Will I use it?*	Use of self-monitoring features	9	✓	Morrison et al [34]; Glasgow et al [54]; Willett et al [48]
*Can I use it?* /*Will I use it?*	Use of positive tone and language	4	✓	Haines-Saah et al [55]
*Can I use it?* /*Will I use it?*	Use of text message	3	✓	Webb et al [27]
*Will I use it?*	Use of action/coping planning	5	✓	Webb et al [27]; Glasgow et al [54]; van Genugten et al [56]
*Will I use it?*	Use of facilitation of social comparison and support	2	✓	Webb et al [27]; Davies et al [57]; Perski et al [58]; Alahäivälä and Oinas-Kukkonen [53]; Xu et al [47]
*Will I use it?*	Use of rewards and incentives	1	✓	Khadjesari et al [59]; Schubart et al [60]; van Genugten et al [56]
All	Use of a combination and a number of features	14		Webb et al [27]; Meade et al [61]

### Identifying Intervention Functions From a Behavioral Analysis

#### Methods

We included a behavioral analysis using the “Behaviour Change Wheel” and COM-B model as outlined by Michie and colleagues [26]. This is a theoretical framework that provides a systematic way of identifying the problem and analyzing the behavioral needs of a target behavior. The “Behaviour Change Wheel” can support intervention design by linking the identified behavioral needs to “intervention functions” through a mechanism of action.

Consistent with the guiding principles and specific design objectives, and for the purpose of this behavioral analysis, we reframed the 3 themes from the Interpretive Descriptive qualitative study into target behaviors: *Should I use it?*—Signing up to SPIN (Table 7); *Can I use it?*—Using SPIN (Table 8); and *Will I use it*?—Returning to SPIN over the 12 weeks (Table 9). The COM-B Model was then used to identify the capability (C), opportunity (O), and motivational (M) components required for each of these behaviors (B) to occur, referring to the specific design objectives. The questions “*what needs to happen for the target behavior to occur?”* and “*is there a need to change?”* facilitated the analysis process [26]. We used this process to identify (or “diagnose”) the relevant COM-B components that need to be addressed for the target behavior to occur (see the Behavioral diagnosis of the relevant COM-B components in Tables7^9^).

**Table 7.** Behavioral analysis of target behavior: signing up to SPIN^a^ (*Should I use it?*) for people living with spinal cord injury who have shoulder pain.

COM-B components^b^	What needs to happen for the target behavior to occur?	Is there a need for change?
Physical capability	Have the physical ability to access SPIN features and functions and use it	No change needed as SPIN will only be suitable for people who can physically access and use it
Psychological capability	Believe they have the capability to use SPIN	*Change* needed as pwSCI^c^ will want reassurance that they have sufficient physical capability to use SPIN and/or that it is suitable for people with their level of physical ability
Psychological capability	Know that exercise can improve pain symptoms (or not make the condition worse)	*Change* may be needed as there may be fears or concerns that exercise could worsen pain symptoms
Physical opportunity	Have a device that can access SPIN	No change needed as SPIN will only be suitable for those people who have devices that can access SPIN
Social opportunity	Know about other pwSCI who have either benefitted from exercise for shoulder pain or are using SPIN	*Change* needed as pwSCI may not know about others who have benefitted from exercise to improve shoulder pain symptoms or who are using SPIN
Reflective motivation	Hold beliefs that exercising will reduce pain symptoms and/or improve activity	*Change* needed as pwSCI may be fearful that exercise may worsen pain symptoms
Reflective motivation	Believe that SPIN has been developed by a credible and trustworthy source	*Change* needed as pwSCI will want to assure themselves that SPIN has been developed by knowledgeable personnel who have experience in SCI^d^ rehabilitation
Automatic motivation	Believe that SPIN will identify those that are suitable (and unsuitable) to use it	*Change* needed as pwSCI will want assurance that SPIN is appropriate for their circumstances and can be tailored for their needs
Automatic motivation	Need to feel that SPIN resonates (with current attitude toward exercise, support situation)	*Change* needed as pwSCI need to feel comfortable that SPIN is right for them at this time
Behavioral diagnosis of the relevant COM-B components	Psychological capability, social opportunity, reflective and automatic motivation need to change for the target behavior to occur	—^e^
Likely ”intervention functions” that link to COM-B	Education (psychological capability, reflective motivation), Training (physical opportunity), Modelling (social opportunity), and Persuasion (reflective motivation, automatic motivation)	—

a SPIN: Shoulder Pain Intervention delivered over the interNet.

b Behavioral diagnosis of the relevant COM-B components: psychological capability, social opportunity, reflective and automatic motivation need to change for the target behavior to occur.

c pwSCI: people living with spinal cord injury.

d SCI: spinal cord injury.

e not applicable.

**Table 8.** Behavioral analysis of target behavior: using SPIN^a^ (*Can I use it?*) for people living with spinal cord injury who have shoulder pain.

COM-B components^b^	What needs to happen for the target behavior to occur?	Is there a need for change?
Physical capability	Have the physical ability to control and manipulate SPIN features and functions and related equipment and setup	*Change* may be needed as pwSCI^c^ will want reassurance that they have sufficient physical capability to use the intervention and/or that the intervention is suitable for people with their level of physical ability
Physical capability	Have the additional support as required	*Change* may be needed with additional support for equipment setup and exercise support
Psychological capability	Believe they have the capability to use SPIN	*Change* needed as pwSCI will want reassurance that they have sufficient physical capability to use SPIN and/or that it is suitable for people with their level of physical ability
Psychological capability	Know how to navigate through the intervention	*Change* needed to clearly provide pwSCI with signposts and information to guide them through
Psychological capability	Know how to perform exercises safely	*Change* needed to ensure appropriate level of exercises is offered and explained to maximize safe exercising and to ensure that the program is responsive to changes in user presentation
Physical opportunity	Have a program that is usable and easy to follow	*Change* needed to ensure SPIN is easy to use and understand
Social opportunity	Haencouragement from peers	*Change* needed to ensure access to a community of users
Reflective motivation	Have confidence in one’s ability to use the intervention program	*Change* needed to provide a sense of ownership and control of the program, with positive reinforcement with use
Reflective motivation	Have belief the intervention will enable achievement of outcomes important to user	*Change* needed as users may not recognize the value of SPIN
Automatic motivation	Have experience of benefit from intervention and sense of progress	*Change* needed to provide consistent exercise opportunities
Behavioral diagnosis of the relevant COM-B components	Physical and psychological capability, physical and social opportunity, and reflective motivation need to change for the target behavior to occur	—^d^
Likely ”intervention functions” that link to COM-B	Training (physical capability, psychological capability), Environmental restructuring (physical opportunity), Modelling (social opportunity), and Persuasion (reflective motivation)	—

a SPIN: Shoulder Pain Intervention delivered over the interNet.

b Behavioral diagnosis of the relevant COM-B components: physical and psychological capability, physical and social opportunity, and reflective motivation need to change for the target behavior to occur.

c pwSCI: people living with spinal cord injury.

d not applicable.

**Table 9.** Behavioral analysis of target behavior: using SPIN^a^ (*Will I use it?*) for people living with spinal cord injury who have shoulder pain.

COM-B components^b^	What needs to happen for the target behavior to occur?	Is there a need for change?
Physical capability	Have the physical ability to control and manipulate SPIN features and functions and related equipment and setup	*Change* may be needed as pwSCI^c^ will want reassurance that they have sufficient physical capability to use the intervention and/or that the intervention is suitable for people with their level of physical ability
Physical capability	Have the additional support as required	*Change* may be needed with additional support for equipment setup and exercise support
Psychological capability	Believe they have the capability to use SPIN	*Change* needed as pwSCI will want reassurance that they have sufficient physical capability to use SPIN and/or that it is suitable for people with their level of physical ability
Psychological capability	Know how to navigate through the intervention	*Change* needed to clearly provide pwSCI with signposts and information to guide them through
Psychological capability	Know how to perform exercises safely	*Change* needed to ensure appropriate level of exercises is offered and explained to maximize safe exercising and to ensure that program is responsive to changes in user presentation
Physical opportunity	Have a program that is usable and easy to follow	*Change* needed to ensure SPIN is easy to use and understand
Social opportunity	Have encouragement from peers	*Change* needed to ensure access to a community of users
Reflective motivation	Have confidence in one’s ability to use the intervention program	*Change* needed to provide a sense of ownership and control of the program, with positive reinforcement with use
Reflective motivation	Have belief the intervention will enable achievement of outcomes important to user	*Change* needed as users may not recognize the value of SPIN
Automatic motivation	Have experience of benefit from intervention and sense of progress	*Change* needed to provide consistent exercise opportunities
Behavioral diagnosis of the relevant COM-B components	Physical and psychological capability, physical and social opportunity, and reflective motivation need to change for the target behavior to occur	—^d^
Likely ”intervention functions” that link to COM-B	Training (physical capability, psychological capability), Environmental restructuring (physical opportunity), Modelling (social opportunity), and Persuasion (reflective motivation)	—

a SPIN: Shoulder Pain Intervention delivered over the interNet.

b Behavioral diagnosis of the relevant COM-B components: physical and psychological capability, physical and social opportunity, and reflective motivation need to change for the target behavior to occur.

c pwSCI: people living with spinal cord injury.

d not applicable.

Next, we mapped these components to established ‘intervention functions,’ using the “Behaviour Change Wheel.” Most relevant “intervention functions” were then identified from the matrix of links between COM-B and intervention functions [26]. The “Behaviour Change Wheel” uses the term “intervention function” in lieu of intervention “type” or “category” since the same intervention feature may address more than 1 function [26].

#### Outcome

Tables7^9^ present the target behavior for each design objective and what (if any) change is needed to occur based on the COM-B components. “Intervention functions” most likely to support behavior change have also been identified. For example, testimonials about positive experiences of using exercise to help with shoulder pain could be a form of modeling (providing an example for people to aspire to) and persuasion (using communication to induce positive feelings or stimulate action). This mapping process allowed each specific design objective to be checked to ensure it was supported by an appropriate “intervention function” and corresponding intervention feature. “Intervention functions” linked to the target behavior have been included in Likely “intervention functions” that link to the COM-B in each of the tables (Tables7^9^). The guiding principles tables (Tables3^5^) provide an overview of how these “intervention functions” map to the design objectives (“Intervention functions” column).

### Step 3: Selection of Intervention Features Applicable to SPIN

#### Methods

The design phase of the PBA involves identifying intervention features and content, guided by the previously formulated guiding principles, to ensure alignment with users’ psychosocial contexts and to enhance relevance, acceptability, and engagement through iterative user feedback [23]. We were able to begin selecting specific SPIN intervention features once the behavioral analysis was complete. The behavioral intervention features previously identified (Table 6) were reviewed. We mapped those that we felt were contextually appropriate against the “intervention functions.” Each was checked to ensure it supported the specific design objectives and the overarching intervention objective. VS completed this process in consultation with coauthors.

#### Outcome

**Figure 3.**
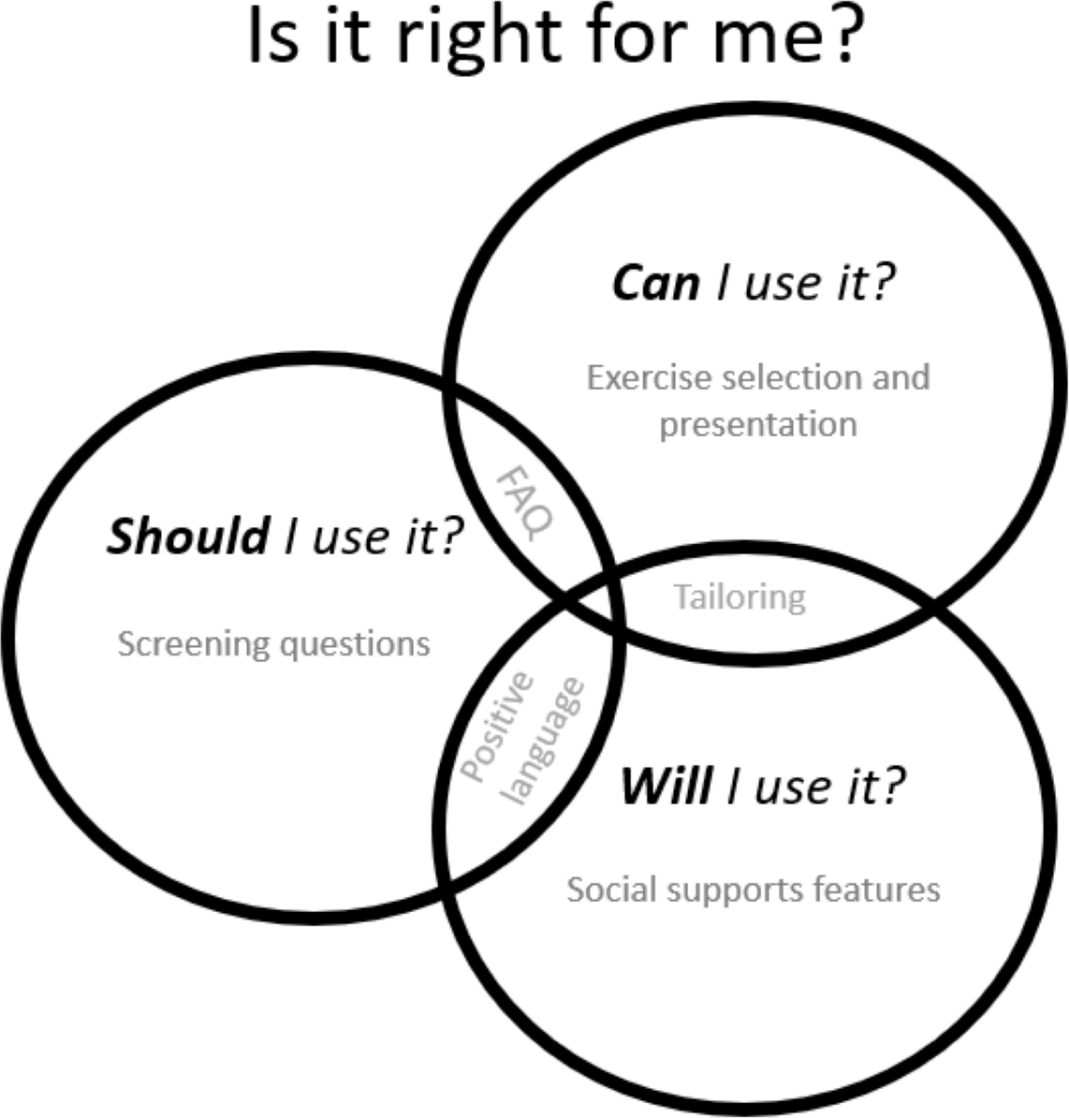
Schematic representation of overlapping intervention features.

Collectively, Tables3^5^ demonstrate a complete representation of the guiding principles of SPIN’s proposed intervention features and functions, mapped back to the design objectives. Some intervention features address more than 1 intervention design objective. These features have been italicized in Tables3^5^. For example, having a forum for frequently asked questions may reduce barriers to starting the intervention and give users the information they need to progress. Having positive, encouraging language can attract users to start using the intervention and motivate them to continue with it. Other intervention features more clearly support only one of the intervention design objectives. Figure 3 schematically presents an example of how overlapping intervention features cohesively support SPIN’s identified design objectives.

### Application of Our Design Steps to Future Intervention Design

We believe that by explicating how we used the PBA in the development of SPIN, we can support others to use the PBA in the design of interventions. Table 10 provides a summary view of our process and includes some questions that we hope will prompt other researchers to consider how they might operationalize the use of PBA in their work. The table provides an overview of key phases of PBA and possible timelines (column 1) and examples from SPIN (column 2), including tools and methods we drew on as complementary to PBA and which we found useful in operationalizing the approach. In column 3, we have included our reflections on the benefits of our approach. The final column has questions that we hope will serve as prompts for researchers and designers when using this approach.

**Table 10.** Operationalizing the person-based approach: our experience and future applications.

1. Key steps in the person-based approach	2. Methods we used to operationalize PBA^a^ steps in the development of SPIN^b^	3. Strengths and opportunities of our approach	4. Questions to consider when planning this step
Step 1 (months 3‐6) Identify key behavioral issues (access), needs (not feeling competent), and challenges the intervention must address	Interpretive Descriptive qualitative study to explore user perspectives of self-guided exercise intervention and what would help or hinder uptake of a self-guided digital exercise intervention. Used probes and images during data collection to help users visualize and provide feedback on possible intervention features.	Drawing on Interpretive Descriptive as a nested study within the PBA process helped to provide a robust framework to capture and make sense of user needs and preferences. Interpretive Descriptive is congruent with the goals of PBA and has the benefit of (1) being oriented toward translation from the outset, (2) prioritizing the production of clinically relevant insights, and (3) flexibility in methods so data collection and analysis could be tailored to the intended use of findings for intervention development.	Who are the users? What is the best way to understand their unique context and specific needs? Are there existing tools and methods available that would be fit for purpose to capture user needs and preferences? How is the information going to be used? How might your approach to capturing needs and preferences be optimized for this intended use? Does data already exist (systematic reviews and qualitative research) that can help inform this step?
Step 2 (months 6-9-12) Creating intervention design objectives that capture what is unique about your intervention and reflect the specifically identified user needs and challenges the intervention needs to address.	Translate themes from the Interpretive Descriptive study into design statements and conditions for success, drawing on the data from each theme.	Helped to reframe the themes into actionable statements. Provided an evidence-based framework to underpin intervention design objectives. Ensured that user needs and preferences will continue to be reflected in the design process.	How are user needs and preferences currently expressed? Can they be used to underpin design objectives in their current form, or do they need some further refinement/transformation?
Step 2 (months 6-9-12) Creating intervention design objectives that capture what is unique about your intervention and reflect the specifically identified user needs and challenges the intervention needs to address.	Two overarching objectives for SPIN were developed from the design statements and conditions of success. It was repeatedly revised, referring to the original research question and design statements, and with input from stakeholders.	Developing 2 overarching objectives, rather than 1, helped to make explicit 2 interrelated but distinct objectives. The intermediary step of developing design objectives from the qualitative study themes ensured that the objectives represent the essence of the needs expressed by the users. Refining with input from stakeholders helped to ensure the objectives remained resonant with the SCI^c^ community. Articulating these objectives at the outset was a useful reference point to keep coming back to for all later design and development phases.	What is(are) the overarching intervention objective(s)? How will you ensure your overarching objective(s) remain(s) grounded by user needs and preferences? Who might need to be involved in the development of intervention objective(s)? How will you know if your intervention objective(s) adequately capture(s) the perspectives of future users?
Step 2 (months 6-9-12) Creating intervention design objectives that capture what is unique about your intervention and reflect the specifically identified user needs and challenges the intervention needs to address.	Specific design objective were identified, drawing on the design statements and overarching intervention objectives. Working definitions were formulated with reference to original data sources and in collaborative discussions as a research team.	The development of specific design statements provided a framework to identify design requirements (system requirements) and intervention features. Investing time to develop the working definitions as a team, with reference to original data sources, was important for clarity and shared understanding.	What process will you use to generate specific design objectives from your overarching design objective(s)? Who might need to be involved in that process? What data sources do you have that you can refer to so you can refine your specific design objectives?
Step 3 (months 6‐12) Select and refine intervention features that support the specific design objectives.	Several methods were used to support the selection and refinement of intervention features for SPIN including: (1) extracting data on intervention features from a previous systematic review on self-guided exercise interventions, (2) reviewing relevant behavioral theory, (3) undertaking a behavioral analysis, and (4) drawing on persuasive system design.	Drawing on a multiplicity of methods in this step (1) ensured an evidence-based and theoretically informed approach and (2) enabled a systematic approach to ensure intervention features were those best suited to the behavioral needs of the SPIN user. A systematic approach to identifying intervention features and mapping them back to design objectives helps to imrove the credibility of intervention design. The outcome was a clear framework for SPIN intervention design that was a useful tool to support communication with design colleagues or software developers who were then bringing SPIN to form.	What data sources are available that can help you identify potential intervention features? What midrange theories are available that can help you identify potential intervention features? Are there existing tools and methods available that would be fit for purpose to help you identify intervention features which respond to user needs and preferences? Of all the potential intervention features, which are most likely to meet the design objective(s)? Who else should be involved in this process? How might you ensure that the outcome of this process can be an accessible and usable framework for others involved in intervention development?

a PBA: person-based approach.

b SPIN: Shoulder Pain Intervention delivered over the interNet.

c SCI: spinal cord injury.

### Ethical Considerations

Ethics approval (Auckland University of Technology Ethics Committee-AUTEC 18/263) and participant consent were received for the earlier work [33] that informed this work.

## Discussion

### Principal Findings

This paper has described how we applied evidence, theory, and person-based approaches in the design of a self-guided digital intervention to help pwSCI manage their shoulder pain. We have detailed the processes of applying the PBA to the design of SPIN.

This builds on Yardley and colleagues’ [23] collection of work. The PBA emphasizes a detailed, qualitative understanding of users’ psychosocial contexts to inform intervention design. It adds value to user-centered design by addressing factors that influence behavior change, beyond just usability. The PBA complements theory- and evidence-based frameworks, such as the “Behaviour Change Wheel” [25] by tailoring interventions to the needs and preferences of specific populations. Despite growing evidence for the use of the PBA framework in intervention design [29-31,62,63], there is little available on its operationalization. To our knowledge, the detailed reporting of each step has not been available before.

In a recent systematic review on the effectiveness of self-guided digital exercise interventions, Stavric et al [21] found that interventions with theoretical underpinning had increased congruence with the intervention features leading to significant positive results. This is supported by findings from McEwan [64] who found that theory-based interventions resulted in more consistent significant improvements in physical activity. The pwSCI and shoulder pain who will use SPIN are likely to have minimal contact with a health care professional. Therefore, successful design required an understanding of how SPIN would meet their needs and how pwSCI would use it in daily life in a self-directed way. Engaging people and evidence in intervention design is supported by a range of researchers and designers [23,34,65]. Using a person-based approach, drawing on evidence from the people who will use the intervention, to derive the behavioral strategies has been shown to be effective in a variety of settings and methods of delivery [18,29,30,66,67].

Despite acknowledgment that interventions supported by theory and evidence maximize outcomes [68,69], there remains a paucity of full intervention description or design disclosure [70-73], making it challenging to explicate the link between theory and evidence and intervention features. A key tension we encountered was the limited availability of detailed examples of how the PBA had been operationalized in practice. This required us to make interpretive decisions when translating PBA principles into design elements, often without clear guidance. Additionally, balancing adherence to the PBA’s iterative, user-focused process with practical constraints such as time, resources, and access to participants posed challenges. These limitations were compounded by the fact that we were largely self-taught in the application of both the PBA and behavioral analysis frameworks.

Michie and colleagues [74] recognized the challenges and lack of clarity around the purported mechanisms by which digital interventions work during an international workshop on developing and evaluating digital interventions to promote behavior change in health. DiLiberto and colleagues [75] support the importance of “insider accounts” of intervention implementation and argue that the same transparent reporting practice should apply to intervention design. Of the 16 self-guided interventions included in our systematic review and meta-analysis conducted in the planning phase [21], only 6 provided any reference to methods used to plan, design, and develop them [19,76-81]. Of these, there was little supporting detail on how the design was carried out and none of the included studies reported exploring the behavioral needs of the users before designing the intervention. Future researchers might benefit from greater transparency and reporting of the design phase, including more practical examples of operationalizing person-based and behavioral approaches. Considerations for mitigating these challenges include allocating sufficient time and resources for user involvement beyond the planning stage, documenting key design decisions, and seeking opportunities for peer collaboration to support methodological alignment and confidence.

### Strengths

We have shown commitment to providing a robust and transparent process in the operationalization of the design phase of SPIN drawing on the PBA approach. This process included explicitly addressing the identified behavioral needs of the users and kept these central throughout the entire design process. The design of SPIN has demonstrated how we used evidence (from existing literature and from a previous Interpretive Descriptive qualitative study) and theory (from behavioral analysis, “Behaviour Change Wheel,” and COM-B) to enhance the person-based process. This explicit and thorough process of planning and designing SPIN has provided a blueprint for intervention development when using PBA. It also addresses many of the limitations in the reporting on the development processes for existing self-guided digital exercise and physical activity-related interventions.

### Limitations

Our operationalization of the PBA design phase reflects our interpretation of the PBA steps through available readings. We acknowledge there may be other perspectives and understandings. However, we believe that it is important to make our experiences visible to build on previous work and support future intervention design. Similarly, we relied on literature and online course instruction for support when we conducted the behavioral analysis using the COM-B. Being self-taught in both the PBA and behavioral analyses may mean that some aspects of our approach are not consistent with the original intent of these approaches. However, this is perhaps an artifact of the knowledge mobilization process, where the application of knowledge can change as knowledge changes hands. By offering transparency in our process, we hope that people can draw their own conclusions regarding the robustness of our approach. The design of SPIN did not include a logic model. Logic models typically include the main intervention components, how they relate to one another, which are meant to produce which effect, and include processes and expected outcomes. However, we did not believe a logic model would have been pragmatically useful as they assume causal relationships which may have restricted our thinking about solutions [82]. Our development process was underpinned by relationship building and community interaction, both of which are complex and require flexibility [83,84].

### Future Steps of SPIN Using the PBA

With the proposed intervention features selected, SPIN wireframes have been constructed. Wireframes are images or screenshots that show how screens of a website or app are structured and how content is arranged. These have provided a visual representation of the product and an opportunity to comment on content, features, and organization without getting distracted by aesthetics. Further participant consultation and design refinement have occurred. Frontend and backend software programming will occur at a later phase. Reporting of these stages will follow in a subsequent publication.

### Conclusion

The design of SPIN has incorporated a deep understanding of the users’ needs and best available evidence by drawing on the PBA design process to maximize chances of engagement and outcomes. This paper has made visible the operationalization of each of the phases and can act as a blueprint to provide guidance to future researchers when using this approach.

## Supplementary material

10.2196/66678 Multimedia Appendix 1 Systematic review data collection template.

## Abbreviations

PBA: person-based approach
pwSCI: people living with spinal cord injury
SCI: spinal cord injury
SPIN: Shoulder Pain Intervention delivered over the interNet
